# The Non-Canonical Wnt/PKC Pathway Regulates Mitochondrial Dynamics through Degradation of the Arm-Like Domain-Containing Protein Alex3

**DOI:** 10.1371/journal.pone.0067773

**Published:** 2013-07-02

**Authors:** Román Serrat, Guillermo López-Doménech, Serena Mirra, Martí Quevedo, Jordi Garcia-Fernàndez, Fausto Ulloa, Ferrán Burgaya, Eduardo Soriano

**Affiliations:** 1 Department of Cell Biology, University of Barcelona, Barcelona, Spain; 2 Developmental Neurobiology and Regeneration Lab, Institute for Research in Biomedicine Barcelona, Parc Científic de Barcelona, Barcelona, Spain; 3 Centro de Investigación Biomédica en Red sobre Enfermedades Neurodegenerativas, Madrid, Spain; 4 Department of Genetics, Faculty of Biology, and Institute of Biomedicine, University of Barcelona, Barcelona, Spain; 5 Fundación CIEN, Vallecas, Madrid, Spain; Northwestern University Feinberg School of Medicine, United States of America

## Abstract

The regulation of mitochondrial dynamics is vital in complex cell types, such as neurons, that transport and localize mitochondria in high energy-demanding cell domains. The Armcx3 gene encodes a mitochondrial-targeted protein (Alex3) that contains several arm-like domains. In a previous study we showed that Alex3 protein regulates mitochondrial aggregation and trafficking. Here we studied the contribution of Wnt proteins to the mitochondrial aggregation and dynamics regulated by Alex3. Overexpression of Alex3 in HEK293 cells caused a marked aggregation of mitochondria, which was attenuated by treatment with several Wnts. We also found that this decrease was caused by Alex3 degradation induced by Wnts. While the Wnt canonical pathway did not alter the pattern of mitochondrial aggregation induced by Alex3, we observed that the Wnt/PKC non-canonical pathway regulated both mitochondrial aggregation and Alex3 protein levels, thereby rendering a mitochondrial phenotype and distribution similar to control patterns. Our data suggest that the Wnt pathway regulates mitochondrial distribution and dynamics through Alex3 protein degradation.

## Introduction

Mitochondria are essential organelles for many biological processes, including respiration, energy production and cell viability. Because of the length of neuronal processes (axons and dendrites) and the function of mitochondria in neurotransmission and neural integration, the correct distribution of these organelles is crucial for neuronal function [1]. In fact, impaired distribution and function of mitochondria and/or mutations in mitochondrial-related motors has been found in neurological diseases, including Parkinson’s, Alzheimer’s, and Huntington’s Disease, as well as in rare disorders such as Charcot Marie-Tooth disease [2]–[4].

In a previous study we provided evidence that proteins encoded by the Eutherian-specific Armcx gene family localize to mitochondria [5]. Furthermore, that study demonstrated that at least one member of this family, Alex3, interacts with the Kinesin/Miro/Trak2 protein complex responsible for mitochondrial trafficking [6]–[8]. Interestingly, this interaction is Ca^2+^-dependent, and Alex3 was found to control mitochondrial aggregation, dynamics and trafficking in neurons [5]. This finding suggests that this Eutherian-specific family of mitochondrial proteins adds a further degree of molecular complexity and regulation to mitochondrial dynamic events in the brains of higher vertebrates.

Members of the Alex protein family (Alex1–3; for Arm-containing protein Lost in Epithelial cancers linked to the X chromosome) were initially described as putative tumor-suppressor genes, as their expression is reduced in several epithelial-derived carcinomas, including lung, prostate, colon, and pancreas cancer [9]. While Alex1 and 2 are widely expressed in numerous tissues, Alex3 is found mainly in the nervous system. Previously, we characterized Alex3 as a gene preferentially expressed in the upper layers of the developing cerebral cortex [10]. That study confirmed the preferential expression of this gene in neural tissue and its developmental regulation. A recent report described Alex3 as a Sox10-interacting protein that localizes in the mitochondria of OBL21 cells and suggested a novel signaling cascade between mitochondria and the nucleus through a Sox10/Alex3 protein complex [11].

Some extracellular signals, synaptic activity, neurotransmitters and growth factors have been reported to regulate the transport and dynamics of mitochondria, thus targeting these organelles to energy-demanding cell territories [12]–[17]. However, very little is known about the molecular mechanism regulating this process and about the extent to which extracellular signals control mitochondrial trafficking and targeting. The Alex3 protein sequence contains 6 Armadillo-like domains, arranged in a unique DUF463 domain, whose function remains unknown [5]. Typically, Armadillo domains are involved in the regulation of Wnt/β-catenin signaling in many cells types and play multiple and important roles in both normal development and in the pathogenesis of numerous diseases, particularly cancer [18]–[20]. Here we describe that the Wnt signaling cascade regulates mitochondrial dynamics by controlling Alex3 protein levels by degradation. Furthermore, our data show that the degradation of this protein is mediated by the Wnt downstream effectors PKC or CKII. Our results support the notion that the Wnt pathway controls mitochondrial dynamics by regulating Alex3 protein levels.

## Materials and Methods

### Plasmid Vectors

*Alex3* 3′-UTR was found in a Substractive Hybridization library [10], and the full sequence was obtained by screening a P0 mouse brain cDNA library (Stratagene). For the generation of Alex3, Alex3-myc and GFP-Alex3ΔNt expression vectors, pBluescript-Alex3 was subcloned into the following expression vectors: pcDNA.3 (Invitrogen), pSecTag-A (Invitrogen) and pEGFP-N3 and pEGFP-C1 (Clontech). For the generation of the constructs Alex3-GFP, AlexΔCt, Alex3(1–200)-GFP, Alex3(1–106)-GFP, Alex3(1–45)-GFP, Alex3(1–41) and Alex3 (1–30), Alex3 was amplified with high fidelity Pfu (Stratagene), and a BamHI restriction site was introduced by using appropriate primers (Forward: 5′-CTATAGGGCGAATTGGGTACCG-3′; and reverse: for Alex3-GFP 5′-GGATCCTTCCTGACTCTTTGGGAACATCC-3′; for Alex3ΔCt-GFP 5′-GGATCCAACATCCTTTCAGTCAGTT-3′; for Alex3(1–200)-GFP 5′-GGATCCAAGCCTGCGCTGATTTTCGG-3′; for Alex3(1–106)-GFP 5′-GGATCCCATCATCATCATCAGACCA-3′; for Alex3(1–45)-GFP 5′-GGATCCATCACCAGAGCCACCCTCA-3′; for Alex3(1–41)-GFP 5′-GGATCCCCAGAGCCACCCTCAGCCA-3′ and for Alex3(1–30)-GFP 5′-GGATCCCTCAGCCATTTTCTCCTTG-3′). All the constructs generated were sequenced with BigDye-Terminator v3.1 (Applied Biosystems). Mitochondrial-targeted DsRed (MitDsRed) was a gift from Antonio Zorzano (IRB Barcelona). pcDNA-Wnt1 and pcDNA-β-catenin S^33^ were a gift from E. Batlle (IRB Barcelona). pcDNA-Fz2-HA, pcDNA-Wnt5a, pcDNA-Wnt11 and pcDNA-Dvl2 were a gift from P. Bovolenta (CBM, CSIC, Madrid).

### Treatment with Wnts and Pharmacological Inhibitors

Recombinant mouse Wnt3a was used at 200 or 400 ng/ml and Recombinant mouse Wnt5a at 400 or 800 ng/ml (both from R&D Systems). LiCl and SB216763 (Sigma Aldrich) were used as inhibitors of GSK3β at 10 mM and 10 µM respectively. MG-132 (Merck-Calbiochem) was used as proteasome inhibitor at 10 µM, SP600125 (Sigma-Aldrich) as Jun-kinase inhibitor at 10 µM and KN62 (Bioscience) as CAMKII inhibitor at 25 µM. Cypermetrin (Biogen) was used as Calcineurin inhibitor at 10 µM, Casein kinase II inhibitor I (Merck-Calbiochem) as CK2 inhibitor at 100 µM, Calphostin C (Merck-Calbiochem) as PKC inhibitor at 1 µM, BAPTA/AM (Merck-Calbiochem) as intracellular calcium chelator at 20 µM, and cycloheximide (Sigma-Aldrich) as protein-synthesis inhibitor at 40 µg/ml. These drugs and reagents were used 4 h after transfection. TPA (Merck-Calbiochem) at 1 µM was used as PKC activator 19 h after transfection. Twenty-four hours after transfection, all the cells were fixed or lysed for immunocytochemistry or Western analysis respectively.

### Cell Culture and Transfection

HEK293 cells were used for all the experiments. Cells were cultured in DMEM medium supplemented with 10% Fetal Bovine Serum (FBS), 2 mM glutamine, 120 µg/ml Penicillin and 200 µg/ml Streptomycin and were maintained at 37°C in the presence of 5% CO_2_. Upon confluence, cells were trypsinized (0.25% w/v) and plated at the desired density. After two days, cells were transfected using Fugene6 (Roche Diagnostics), following the manufacturer’s instructions, and using a 1∶1 DNA ratio (or as indicated) when two constructs were transfected. Cells were processed as required 24–36 h after transfection.

### Immunocytochemistry

HEK293 cells were fixed in 4% paraformaldehyde. After fixation, they were permeabilized with Triton X-100 in PBS and blocked with blocking buffer (10% FBS (Roche Diagnostics), 0.2 M glycine, 0.1% Triton X-100 and 0.05% Deoxicolic acid in PBS-2% gelatin) for 1 h at room temperature. To label the cells, the following antibodies or dyes were used: rabbit anti-Alex3 (1∶300) [5], rabbit anti-GFP (1∶500, Invitrogen), mouse anti-β-catenin (1∶500, Beckton Dickinson) in blocking buffer for 2 h and with the corresponding secondary antibodies labeled with fluorochromes (Alexafluor 546 or 488, Invitrogen, Carlsbad, CA). Nuclei were stained with bisbenzimide (Hoechst-33342). When necessary, mitochondria labeling was carried out by incubation with the mitochondrion-selective dye MitoTracker Orange CM-H2TMRos (1∶2000, Molecular Probes, Invitrogen) in culture medium for 30 min at 37°C prior to cell fixation. All samples were then mounted on Mowiol. Because Alex3 protein colocalizes with the mitochondrial network in HEK293T cells [5], even after Wnt treatments (not shown), we routinely used Alex3 immunolabeling to monitor and analyze mitochondrial networks.

### Protein Cell Extracts and Western Blot

HEK293 cells were obtained and lysed in Laemmli Buffer (LB) at 98°C for 5 min. 20 µg of protein for each sample was loaded and run in polyacrylamide gels at 100 V. Transfer to nitrocellulose membranes was performed in 120 mM glycine, 125 mM Tris, 0.1% SDS, and 20% methanol at 35 V o.n. Membranes were then blocked in 5% powder milk in TBS and incubated with primary antibodies anti-Alex3 (1∶2000), anti-GFP (1∶1000, Invitrogen), anti-β-catenin (1∶1000, Beckton Dickinson), anti-HA (1∶1000, Invitrogen). Anti-actin (1∶1000, Chemicon, Temecula, CA) or β-tubulin (1∶50.000, Atom) were used as a loading control. Secondary antibodies coupled to HRP were used diluted 1∶2500 in TBS containing 5% powder milk. Labeling was visualized with ECL plus (Amersham Pharmacia Biotech).

### Quantification Analysis

For quantification of mitochondrial phenotypes, cells were classified as “Normal” (cells with an even distribution of mitochondria forming a dense meshwork), “Aggregated” referred to mitochondrial phenotypes with clustered mitochondria near the perinuclear zone, and “Mild-phenotype” for intermediate phenotypes [5]. Between 113 and 344 cells from 2 independent experiments were quantified for each condition. For Western Blot quantification, “Gel-Pro Analyzer” Software was used. The IOD value was normalized with respect to the “Alex3 control” value and results were shown below the images. Results below “0.05” were considered as “0”.

### Live Imaging Analysis

HEK293T cells were seeded onto Poly-D-lysine-coated *Fluorodish* plates (World Precision Instruments, Inc) transfected with Alex3-GFP, MitDsRed or Wnt1 (as above) and filmed 24 h later using a Leica TCS SP2 confocal microscope (Leica Microsystems) equipped with a 63x immersion oil objective. Treatment with TPA was used as described above. All the cultures were kept at 37°C using a heating insert on the microscope stage and an incubating chamber allowing circulation of a controlled CO_2_ (5%)-air heated mixture for the control of pH. Time-lapse series of image stacks composed of 5 images (512×512 px) were taken every 6 sec over 8 min using Leica Confocal Software (Leica Microsystems). Further image processing and video compilation (7 frames per sec) and edition was done with ImageJ software (version 1.43K, NIH, USA).

## Results

### The N Terminus Region of Alex3 is Necessary and Sufficient for Mitochondrial Targeting and Aggregation

**Figure 1:**
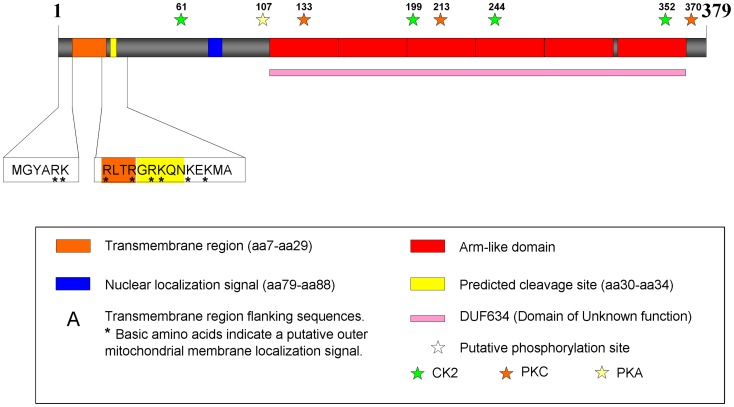
Schematic representation of Alex3 protein. Predicted domains are annotated on the basis of databases such as Pfam, Smart or Wolfpsort and bibliographic references. The stars show the position of putative phosphorylation sites in serine or threonine residues by CK2, PKC and PKA kinases.

**Figure 2:**
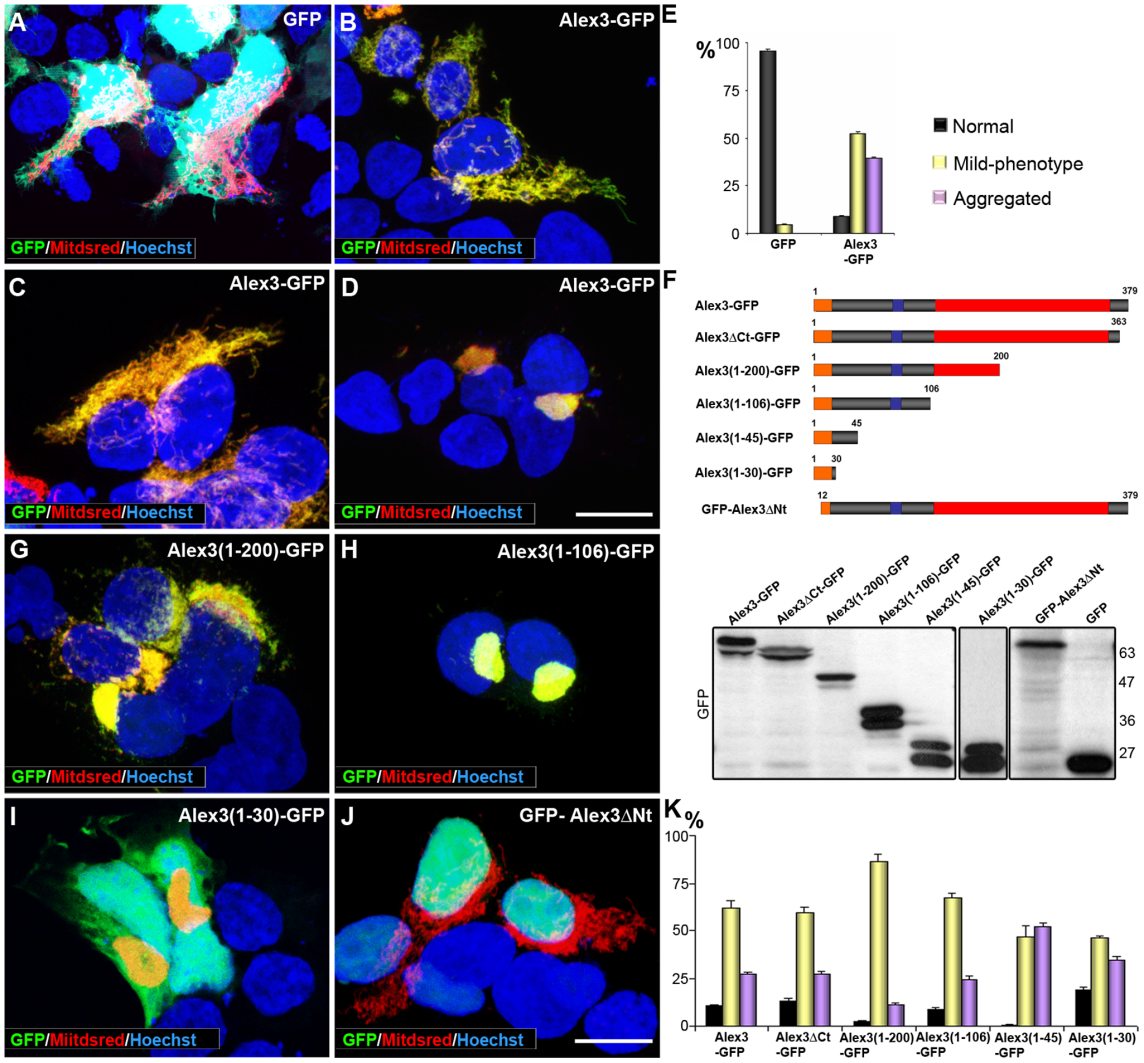
The N-terminal domain of Alex3 is sufficient to induce mitochondrial aggregation. (**A–D**) Overexpression of Alex3-GFP (green) in HEK293T cells induces severe alterations of the mitochondrial network when compared with the expression of control GFP (**A**). (**B**) Illustrates an Alex3-transfected cell displaying normal mitochondrial morphology; (**C,D**) Alex3-overexpressing cells showing mild aggregating phenotypes (**C**) and severe aggregating mitochondrial phenotypes (**D**); Alex3 protein was visualized in green, mitochondria in red (MitDsRed), and nuclei were labeled with bisbenzimide (blue). (**E**) Quantification and graphical representation (mean ± standard deviation) of mitochondrial phenotypes in control (GFP) and Alex3-GFP-overexpressing cells. (**F**) Top: Scheme of the Alex3-GFP deletion constructs used for transfection. Bottom: Western Blot showing representative truncated Alex3-GFP constructs at the predicted protein sizes. (**G–J**) Photomicrographs illustrating that expression of the Alex3(1–200)-GFP (**G**), Alex3(1–106)-GFP (**H**) and Alex3(1–30)-GFP (**I**) constructs leads to mitochondrial aggregation; in contrast, deletion of the first N terminal 12 aa (GFP-Alex3ΔNt) targets Alex3 protein to the nucleus (**J**). Note that the 30 aa N-terminus deletion construct has a truncated outer mitochondrial membrane localization sequence, which may interfere with its mitochondrial targeting, thereby leading to nuclear localization. (**K**) Quantification and graphical representation (mean ± standard deviation) of mitochondrial phenotypes in HEK293T cells after transfection with several truncated Alex3-GFP constructs; the data show that all the constructs containing the N terminal region cause mitochondrial aggregation. Alex3 protein was visualized in green (GFP), mitochondria in red (MitDsRed) and nuclei in blue (bisbenzimide). Scale bar: 10 µm.

Alex3 is a 379-aa protein containing several regions and motifs, including 6 Arm-like domains conforming a DUF463 domain (aa 110–363), a nuclear localization signal (aa 89–98), and an N-terminal region containing a transmembrane domain (aa 7–29) and a putative outer mitochondrial membrane targeting signal (Figure 1). Gavel and von Heijne’s method [21] also detected a predicted cleavage site for mitochondrial presequence translocase (aa 30–34). These predicted mitochondrial-related protein sequences are consistent with the preferential mitochondrial localization of Alex3 protein [5], [11]. To characterize the Alex3 protein regions required for mitochondrial targeting, we generated several Alex3 constructs with deletions at the C-terminal region and tagged them with GFP. In agreement with a previous study [5], transfection of Alex3 cDNA in HEK293AD cells led to mitochondrial aggregating phenotypes, which varied between mild aggregation mitochondrial phenotypes (mild-phenotype), in which individual mitochondria were still visible (52% cells), and strong aggregating phenotypes, which led to single, large mitochondrial aggregates located close to cell nuclei (39% cells) (Figure 2A–E). For these experiments, we indistinctly used the mitochondrial markers MitDsRed and Mitotracker, which labeled the mitochondrial network efficiently and in a similar manner; furthermore, both markers fully co-localized with Alex3 (Figure S1). Transfection with all the C-terminus-deleted Alex3 cDNA constructs, including Alex3ΔCt-GFP, Alex3(1–200)-GFP, Alex3(1–106)-GFP, Alex3(1–45)-GFP, yielded similar mitochondrial phenotypes (Figure 2F–K and not shown). Interestingly, we found that even the smaller construct, containing only the first 30 N-terminus aa (Alex3(1–30)-GFP), resulted in identical mitochondrial phenotypes. This observation thus indicates that this N-terminal region is sufficient for both mitochondrial targeting and aggregation (Figure 2I). Conversely, transfection with an Alex3 cDNA lacking only the first N-terminal 12 aa prevented mitochondrial targeting (and aggregation), thereby indicating that the N-terminal region, where the mitochondrial targeting signal is predicted, is required to tether Alex3 protein to mitochondria (Figure 2J). Control transfections, including pcDNA and pEGFP-N3 plasmids, did not result in mitochondrial aggregation.

### The Wnt Pathway Regulates Alex3 Protein Levels and Mitochondrial Aggregation

**Figure 3:**
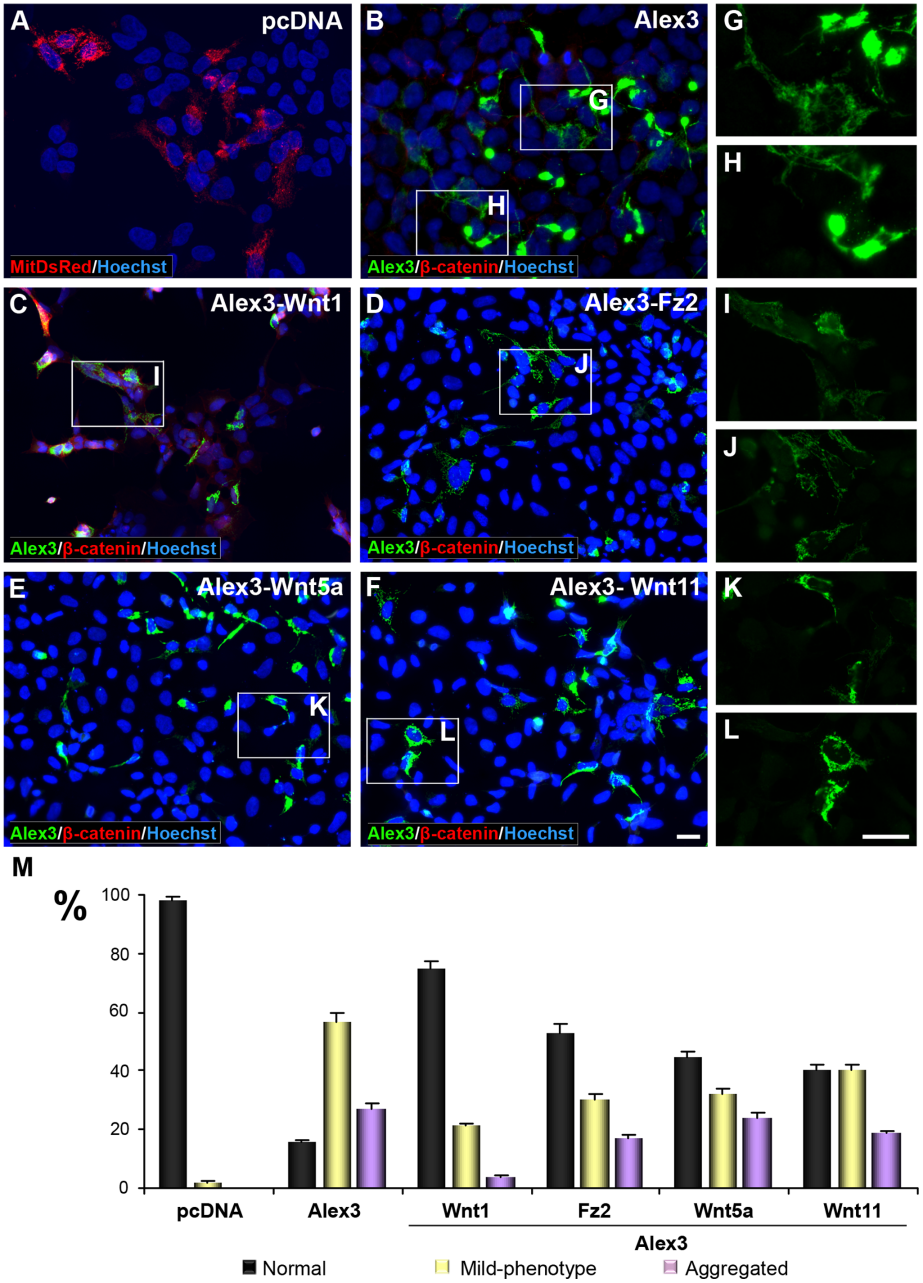
Wnt/Frizzled signaling restores the normal mitochondrial phenotype in Alex3-overexpressing cells. (**A–F**) Co-expression of Alex3 (green) and several members of the Wnt/Frizzled signaling pathway; Wnt1, Fz2, Wnt5a and Wnt11 reverse the aggregated mitochondrial phenotypes induced by Alex3 overexpression in HEK293AD cells. (**G–L**) High magnifications of boxed areas shown in (**A–F**). Note that the aggregated phenotype induced by the expression of Alex3 (G,H) is reversed by the co-expression of different members of the Wnt pathway (I–L). (**M**) Quantification and graphical representation (mean ± standard deviation) of mitochondrial phenotypes resulting after transfection with distinct Wnts and Fz2, demonstrating different degrees of mitochondrial aggregation reversion by the constructs used. Alex3 protein was visualized in green, control mitochondrial distribution (**A**) in red (MitDsRed), β-catenin in red (**B–F**) and nuclei in blue (bisbenzimide). Scale bar: 10 µm.

The Alex3 protein sequence contains 6 Arm-like domains arranged at the C-terminal region (Figure 1). Some previous studies have proposed a putative link between mitochondrial proteins and the Wnt/β-catenin pathway [22], [23]. We thus examined whether Wnt proteins affected the phenotypes induced by Alex3. We found that the mitochondrial aggregating phenotypes caused by the expression of this protein were dramatically reversed by co-transfecting HEK293AD cells with Wnt1 cDNA, which produced a disaggregation phenotype in contrast to HEK293AD cells transfected with Alex3 alone (Figure 3A–C and Figure S2). We next analyzed whether non-canonical Wnt signaling components affected Alex3-induced mitochondrial phenotypes. We observed that co-transfection of Alex3 with the receptor Fz2, Wnt5a or Wnt11 cDNAs had intermediate effects on mitochondrial disaggregating phenotypes under our transfection conditions, in comparison with the canonical Wnt1 protein (Figure 3D–F and Figure S2 and S3). These data were reinforced by experiments in which recombinant Wnt5a led to decreased cellular levels of Alex3 and mitochondrial disaggregation (Figure S4). In contrast, treatment with another canonical Wnt member, Wnt3a, did not result in disaggregation of Alex3-induced mitochondrial phenotypes (Figure S5). High magnifications of the Alex3-transfected cells (Figure 3G–L) and a quantitative evaluation of the mitochondrial phenotypes seen after transfection or incubation with the several Wnt members and signaling components substantiate this notion (Figure 3M). Moreover, the mitochondrial localization of Alex3 was maintained in all the above conditions (Figure S2 and S4).

**Figure 4:**
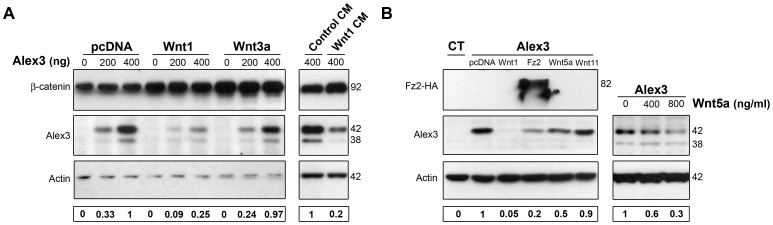
The Wnt/Frizzled pathway induces the degradation of Alex3 protein. (**A**) WBs showing that Wnt1 co-transfection (left) and incubation with Wnt1-conditioned media (CM, right), but not treatment with Wnt3a (200 ng/ml) (middle), induces the degradation of Alex3 protein. (**B**) WB showing that co-transfection with Wnt1, Fz2, Wnt5a and Wnt11 lead to different reductions in Alex3 protein levels (left). Recombinant Wnt5a also induces Alex3 degradation in a concentration-dependent manner (right).

During the course of the above experiments, we noted that Alex3 immunofluorescence signals decreased upon treatment with Wnt1 and Fz2 (see Figure 3C,D). To support this observation, we performed Western Blot analyses on transfected HEK293AD cell lysates. First, we confirmed that transfection with Wnt1 cDNA or treatment with recombinant Wnt3a protein or incubation with Wnt1 protein stabilized β-catenin levels, thus indicating that these extracellular factors were functional (Figure 4A). Next, we measured Alex3 protein levels by Western Blot. As shown in Figure 4A, while Wnt1 transfection or incubation led to a dramatic decrease in Alex3 protein levels, Wnt3a treatment at 200 ng/ml had no effect on this parameter. Furthermore, we found that Fz2 caused a dramatic decrease in Alex3 levels; in contrast, Wnt5a and Wnt11 produced a mild decreased in Alex3 protein levels, which accounted for ∼20–50% (3 independent experiments; Figure 4B). Moreover, we did not observe signs indicative of cell death (e.g., pyknotic cells in cultures) in any experiment or condition. Taken together, these findings indicate that Wnt1, and to a lesser extent other Wnt members, lead to a decrease in Alex3 protein levels, which in turn results in an almost complete reversion of the Alex3-induced phenotypes on mitochondrial aggregation.

### Alex3 Levels are not Regulated by Canonical Wnt/β-catenin Downstream Signaling Components

**Figure 5:**
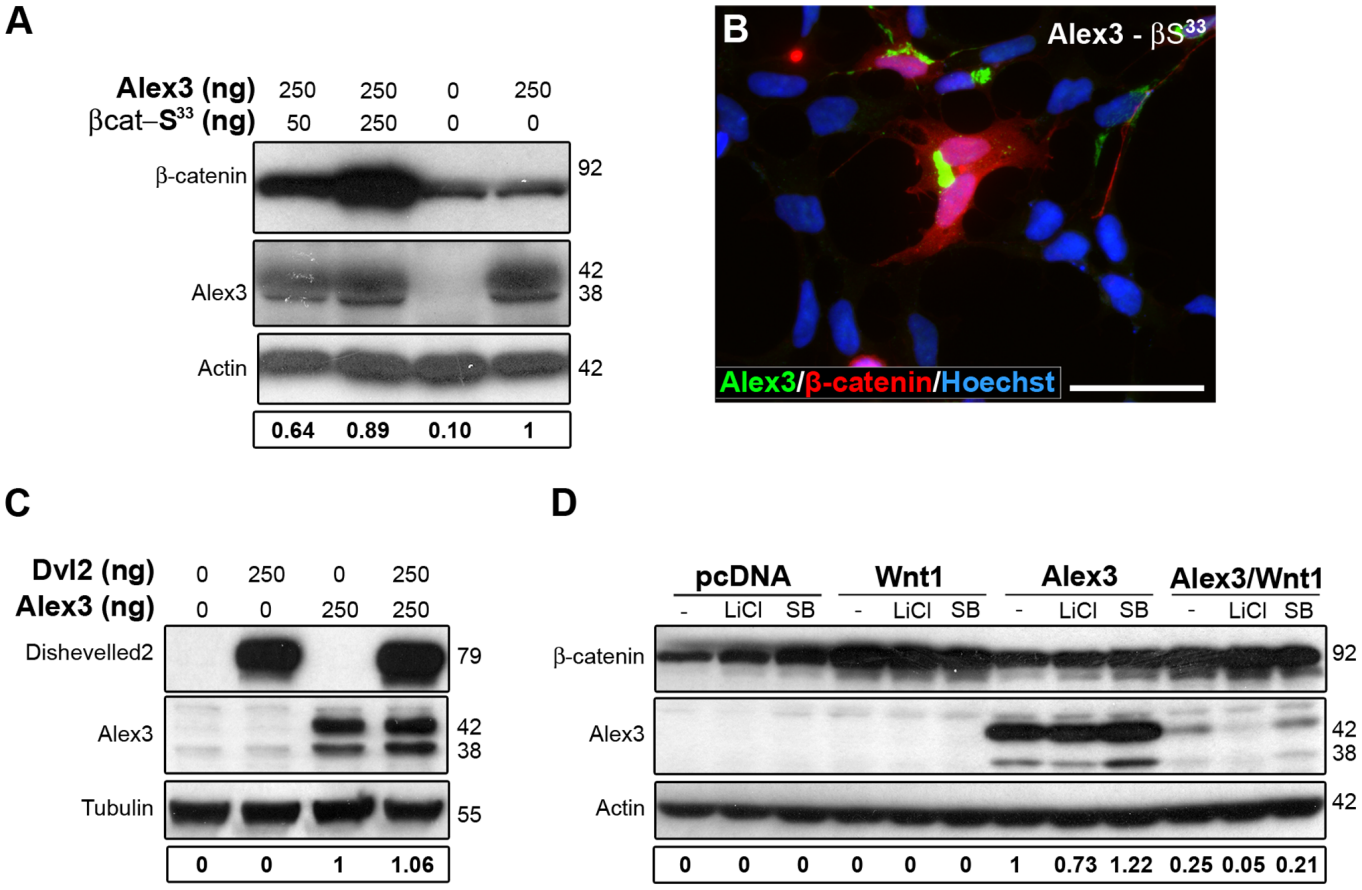
Alex3 degradation is independent of the canonical Wnt/β-catenin pathway. (**A,B**) Constitutively active β-catenin (red) neither induces Alex3 protein degradation, as seen in WB (**A**), nor reverts the aggregated mitochondrial phenotypes induced by Alex3 overexpression (green in **B**). Nuclei were visualized in blue (bisbenzimide) (**B**). (**C,D**) Neither co-transfection with Dvl2 (**C**) nor the inhibition of GSK3β with 10 mM LiCl or with 10 µM SB212763 (**D**) induces Alex3 protein degradation. Wnt1 transfection was used as a control for Alex3 degradation. Scale bar: 10 µm.

We next addressed whether components of the canonical Wnt pathway, which are known to activate the β-catenin pathway, affected Alex3 protein levels. First, we found that co-transfection of Alex3 with constitutively active β-catenin S33A [24] did not result in Alex3 degradation (Figure 5A). In agreement with these results, individual HEK293AD cells with highly stabilized β-catenin displayed Alex3-induced mitochondrial aggregating phenotypes which were similar to those observed after transfection of Alex3 cDNA alone (see above and Figure 5B). Furthermore, transfection with Disheveled2, which also activates the canonical Wnt/β-catenin pathway, was also ineffective at preventing a decrease in Alex3 protein levels (Figure 5C).

Finally, incubation with two inhibitors of GSK3β activity, namely LiCl or SB216763, did not prevent Wnt1-induced Alex3 degradation (Figure 5D). Taken together, these experiments indicate that activation of several intracellular components of the canonical Wnt/β-catenin pathway is not sufficient to cause Alex3 degradation or to alter the degradation of Alex3 induced by Wnt1 treatment.

### PKC Regulates Alex3 Degradation

**Figure 6:**
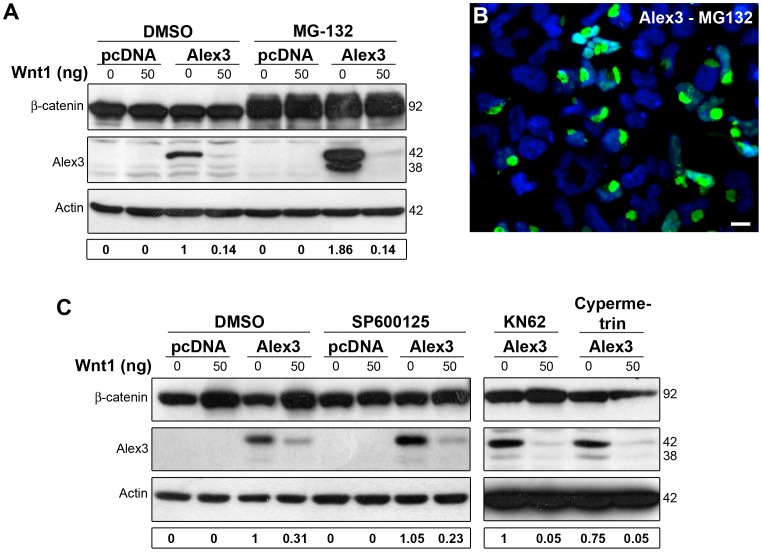
Alex3 degradation by Wnt1 is independent of the proteasome, JNK, CAMKII and Calcineurin pathways. (**A**) Proteasome inhibition with 10 µM MG-132 treatment blocks the normal turnover of Alex3 protein but not its Wnt1-induced degradation. (**B**) Numerous Alex3-overexpressing HEK293AD cells treated with the proteasomal inhibitor MG132 show the most severe mitochondrial aggregating phenotype. (**C**) Inhibition of JNK with 10 µM SP600125 (downstream effector of the Wnt/PCP pathway), CAMKII with 25 µM KN62 or Calcineurin with 10 µM Cypermetrin (downstream effectors of the Wnt/Ca^2+^ pathway) do not induce Alex3 protein degradation. Scale bar: 10 µm.

We next tested whether the degradation of Alex3 triggered by Wnt1 was dependent on the proteasome. First, we demonstrate through cycloheximide assays that Alex3 Wnt/PKC-dependent reduction levels is due to protein degradation (Figure S6). We then found that inhibition of the proteasome with MG-132 incremented Alex3 protein in the absence of Wnt1 (Figure 6A,B). However, the same inhibitor did not alter the degradation of Alex3 induced by Wnt1 (Figure 6A).

The above results suggested that the Wnt signaling mechanisms that lead to Alex3 degradation are upstream of the above Wnt signaling components examined (β-catenin, Disheveled2 and GSK3). We next tested the impact of additional, non-canonical signaling pathways that are activated by the Wnt cascade, such as the Wnt/Planar Cell Polarity (PCP) Jun kinase pathway and the Wnt/Ca^2+^ pathway [18]–[20]. However, inhibition of Jun kinase (with SP600125), CAMKII (KN62) and Calcineurin (Cypermetrin), all three downstream components of the PCP and Ca^2+^ pathways, did not reduce the degradation of Alex3 protein caused by Wnt1 incubation (Figure 6C).

**Figure 7:**
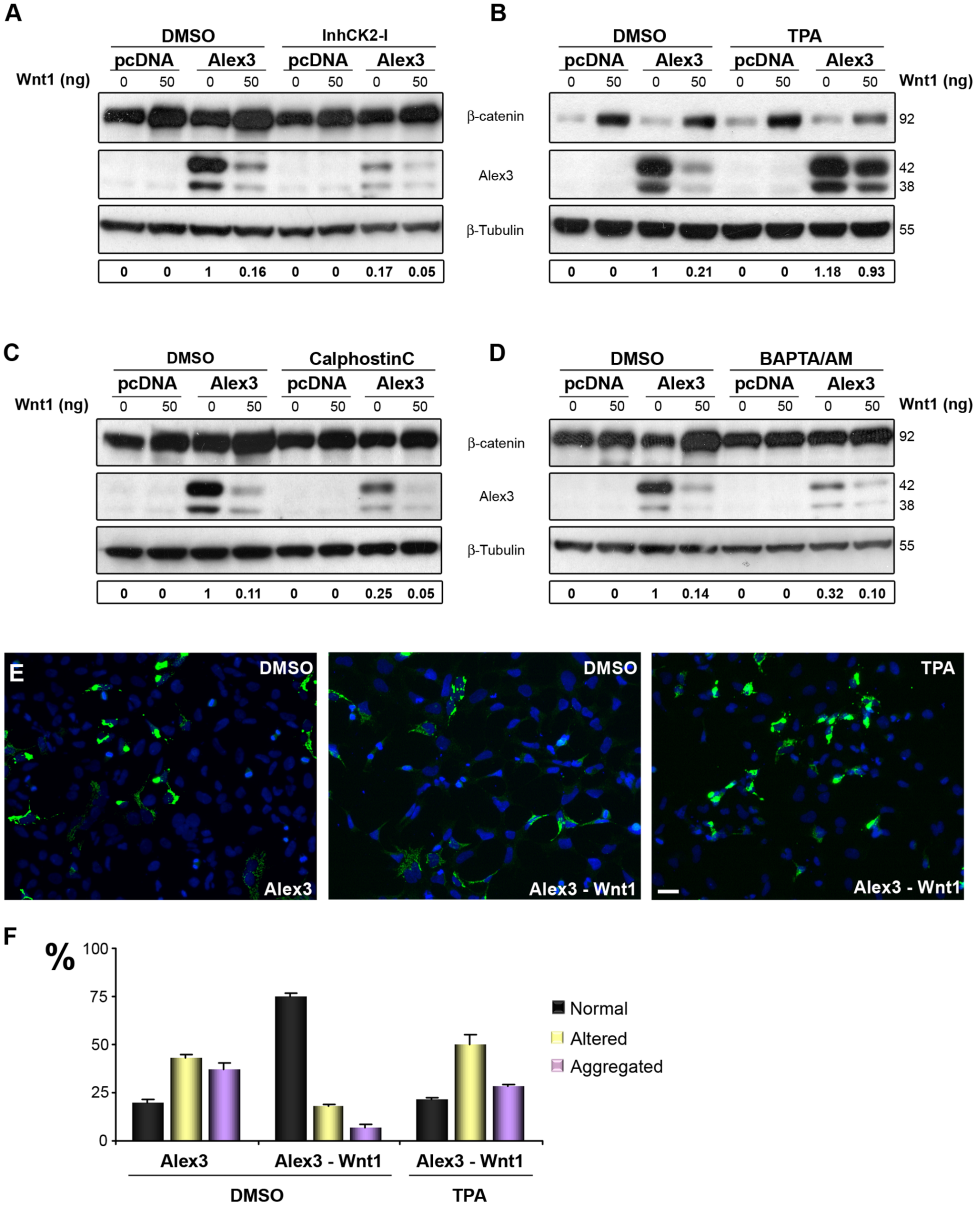
PKC and CKII phosphorylation protects against Wnt/Frizzled degradation of Alex3. (**A**) Inhibition of CKII (with 100 µM casein kinase II inhibitor I), downstream effector of the Wnt signaling pathway, is sufficient to trigger Alex3 degradation. (**B**) In contrast, PKC activation with 1 µM TPA protects against Wnt1-induced degradation of Alex3 protein. (**C**,**D**) Inhibition of PKC (with 1 µM Calphostin C) and treatment with 20 µM BAPTA/AM, an intracellular calcium chelator, also reproduces Wnt1 degradation. (**E**) Photomicrographs demonstrating that treatment with TPA prevents Alex3 degradation induced by Wnt1 and the reversion to normal mitochondrial phenotypes. (**F**) Quantification and graphical representation (mean ± standard deviation) of mitochondrial phenotypes in HEK293AD cells in the conditions shown in (**E**); note that incubation with TPA prevents the rescue of mitochondrial phenotypes induced by Wnt1. Scale bar: 10 µm. The quantification of Alex3 protein levels is shown at the bottom.

Phosphorylation/dephosphorylation often targets proteins to degradation [24]. The Alex3 protein sequence has several putative phosphorylation sites for CK2, PKA and PKC, all of them downstream effectors of the Wnt pathway (Figure 1). We thus tested whether the inhibition of these kinases affected Alex3 degradation. CK2 inhibition (using CK2 inhibitor-1) reduced the levels of Alex3 protein and increased the Wnt1-induced degradation of this protein (Figure 7A), thereby suggesting that dephosphorylation of Alex3 activates its degradation. Furthermore, we found that activation of PKC by TPA abolished the Wnt1-mediated degradation of Alex3 (Figure 7B). Conversely, inhibition of PKC by Calphostin C or by using the Ca^2+^ intracellular chelator BAPTA/AM slightly enhanced the Alex3 degradation induced by Wnt1 (Figure 7C,D). Interestingly, blockade of PKC in the absence of Wnt1 was sufficient to reduce Alex3 protein levels. Moreover, we observed that TPA incubation was sufficient to prevent the mitochondrial disaggregation phenotype induced by Wnt1 in HEK293AD cells (Figure 7E,F).

We further examined whether the Alex3(1–45)-GFP and Alex3(1–106)-GFP constructs, which lack putative PKC phosphorylation sites (Figure 1), responded to Wnt1. Co-transfection of these constructs with Wnt1 or treatment with the PKC inhibitor Calphostin C did not result in reduced Alex3 protein levels, in comparison with full-length Alex3-GFP (Figure S7). Together, these findings suggest a role of PKC in the control and degradation of Alex3 protein in both Wnt1-dependent and -independent (e.g., Calphostin C experiments) manners.

### Wnt1 Regulates Alex3-dependent Mitochondrial Dynamics through a PKC-dependent Mechanism

**Figure 8:**
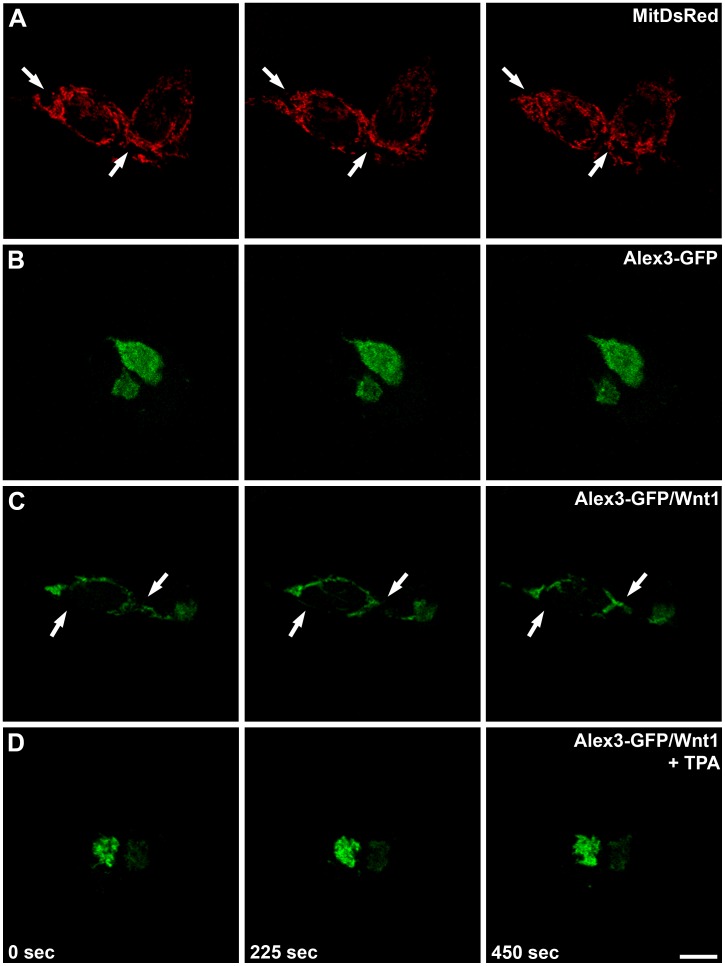
Wnt1 increases mitochondrial motility and dynamics. Series of representative confocal images, taken every 225 sec, from live HEK293T cells overexpressing the mitochondrial tagged protein MitDsRed (**A**)**,** Alex3-GFP fusion protein (**B**), or Alex3-GFP and Wnt1 cDNAs (4∶1) (**C,D**). In (**D**) TPA treatment was used to activate PKC. Arrows identify areas with highly dynamic mitochondria. While mitochondrial motility is high in control (**A**) and Alex3-GFP/Wnt1 (**C**) conditions, it is severely reduced in Alex3-GFP-overexpressing cells (**B**) and in Alex3-GFP/Wnt1/TPA-treated cells (**D**). (See also Videos S1, S2, S3, and S4). Scale bar: 10 µm.

In a previous study we showed that Alex3 overexpression in HEK293 cells leads to mitochondrial aggregation and reduced mitochondrial motility [5]. To examine whether Wnt1 alters these phenotypes, we imaged mitochondrial motility by performing video recordings (n = 3–4 movies per group). Representative static images over time and movies are illustrated (Figure 8 and Supporting Information). Control HEK293T cells displayed a dense meshwork of mitochondria which moved dynamically, similar to previous observations [25] (Figure 8A and Video S1). Overexpression of Alex3 resulted in the progressive aggregation of individual mitochondria in large clusters near the nucleus. These organelles exhibited dramatically reduced dynamics and motility (Figure 8B and Video S2). Cultures transfected with Wnt1 alone or treated with TPA were first examined to assess that most Alex3-transfected cells displayed the phenotypes described above. In agreement with the above data, transfection with Wnt1 cDNA decreased Alex3 protein levels in HEK293T cells; moreover, Wnt1 activation increased mitochondrial motility and dynamics, as observed in the video recordings (Figure 8C and Video S3). Finally, this reversed mitochondrial phenotype was abolished by the treatment of HEK293T cells co-transfected with Alex3 and Wnt1 with the PKC activator TPA. This phenotype again exhibited large mitochondrial clusters with reduced motility (Figure 8D and Video S4). Taken together, these findings suggest that Wnt1 controls not only Alex3 protein levels but also mitochondrial dynamics and motility in a PKC-dependent manner.

## Discussion

Mitochondrial trafficking and dynamics is essential for cell respiration and thus for cell viability [26]. Recent studies have shown that mitochondrial dynamics in neurons (transport and membrane fusion-fission) is a highly regulated process largely mediated by Kinesins, the GTPases Mitofusins1–2 and Miro1–2, and the adaptor protein Trak2 [27]–[29]. We have recently shown that Alex3 protein belongs to the KIF5/Miro/Trak2 protein complex and that its overexpression or knock-down alters mitochondrial trafficking in neurons [5]. Interestingly, Alex3 belongs to a novel family of proteins that controls the distribution, aggregation and dynamics of mitochondria and that is evolutionarily specific for Eutherian mammals [30]. It was predicted that the Armcx gene cluster arose by retrotransposition from a single Arm-containing gene (Armc10) exclusive to and present in all vertebrates and that also regulates mitochondrial trafficking [5]. It is thus likely that the proteins encoded by the Armcx gene cluster add further molecular complexity to the regulation of mitochondrial trafficking, specifically in the nervous system of higher vertebrates.

In addition to mitochondrial localization and putative mitochondrial targeting sequences, Alex3 protein contains six Arm-like repeats, arranged in a single DUF463 domain, at the C-terminal region. On the basis of this enrichment in Arm-like domains, we propose that Alex3 functions and thus Alex3-dependent mitochondrial dynamics are regulated by the Wnt/β-catenin signaling cascade. The present data show that overexpression of Alex3 leads to mitochondrial aggregation and decreased mitochondrial dynamics and trafficking. These effects were reversed upon Wnt1 treatment, which led to reduced Alex3 protein levels and concomitantly rendered mitochondrial morphology and dynamics similar to control phenotypes. In our experimental conditions, a similar, though less marked effect, was found with co-transfection with the receptor Fz2 or the Wnt members Wnt5a and Wnt11. Interestingly, neither transfection with a constitutively active β-catenin S33A cDNA or a Dvl2 cDNA, or incubation with GSK3β enzymatic inhibitors (LiCl and SB216763) had effects on Alex3 protein levels or mitochondrial aggregation. These findings suggest that these typical components of the Wnt/β-catenin pathway are not required for the Wnt1-dependent regulation of Alex3-mitochondrial phenotypes.

In addition to the canonical β-catenin pathway, the Wnt pathway signals through several non-canonical cascades, including the kinases CKII and PKC. Originally, Wnts were classified as canonical (such as Wnt1 or Wnt3a) or non-canonical (Wnt5a or Wnt11) depending or their capacity to induce secondary axis in *Xenopus* embryos [31] or to transform the mammalian epithelial cells C57MG [32]. However, recent studies have shown that several Wnts act in both pathways because of the complexity of cellular contexts. Thus they have specific interactions with various co-receptors and activate distinct signaling molecules, making it difficult to keep such a Wnt classification [33], [34]. Thus, the canonical and non-canonical Wnt pathways can be activated by same Wnt members.

Our results show that the PCP pathway and the downstream Wnt/Ca^2+^-dependent effectors CAMKII and Calcineurin do not affect Alex3 protein levels or Alex3-dependent mitochondrial morphology. In contrast, inhibition of CKII was shown to increase Wnt1-dependent Alex3 degradation and, conversely, activation of PKC abolished Wnt1-dependent Alex3 protein levels and mitochondrial morphology and dynamics. These data suggest that these 2 kinases are involved in the regulation of Alex3 protein levels. Although we cannot confirm direct phosphorylation by these kinases, this notion is consistent with the prediction of several CKII and PKC phosphorylation sites in the Alex3 sequence. Moreover, Alex3 fragments lacking these phosphorylation sites are not affected by either Wnt1 transfection or by Calphostin C treatment. Interestingly, up to 5 of these phosphorylation sites fall within the DUF463 domain, which contains six Arm-like domains (Figure 1). Another extracellular factor, EGF, has been shown to phosphorylate Alex3 at residues that are putative targets for CKII [35]. Our data suggest that Alex3 de-phosphorylation targets Alex3 protein to a proteasome-independent degradation pathway. The observation that Alex3 overexpression causes a perinuclear aggregation similar to that described in mitophagy events [36] raises the possibility that this protein is degraded by this pathway.

Interestingly, we found that activation of PKC protects Alex3 from Wnt-dependent degradation. Although PKC has been described to be activated by non-canonical Wnt signaling, our findings could be explained on the basis of the complex regulation of this kinase. Several PKC isoenzymes are activated by Wnt members and are thus translocated to the cell membrane [37]. It is well known that PKC localizes to several cell compartments (including mitochondria) and that this differential localization regulates its activity [38], [39]. Our data suggest that PKC translocates to the cell membrane after Wnt activation, thereby reducing PKC mitochondrial levels and consequently preventing PKC/Alex3 interaction, thus enhancing Alex3 degradation. Thus, although future experiments are required to unravel the exact signaling cross-talk between PKC and the Alex3 pathway, our results show very consistent effects of Wnts and PKC on Alex3 protein stability.

The role of PKC in the non-canonical Wnt pathway is unclear. However, several studies have shown the participation of this pathway in the regulation of processes such as cell proliferation, differentiation, and apoptosis [40]–[44]. Some isozymes of PKC, such as PKCδ, have been related to specific processes such as convergent extension movements during embryonic gastrulation [37], and atypical PKCs have been implicated in neuronal polarization [45]. Finally, Wnt-regulated PKC has been linked to the progression of cancer, where it may mediate Wnt-dependent cell motility, invasion and metastasis [46]–[49]. Given that Alex3 protein was initially described as a putative tumor suppressor factor and is deleted in several epithelial carcinomas [9], and that the Wnt/β-catenin pathway plays a prevalent role in cancer initiation and tumoral growth [20], [50], future analyses are required to unravel the exact contribution of Alex3 to these pathological processes and the regulation of this protein by the Wnt pathway.

Our results support the notion that Alex3 protein leads to mitochondrial aggregation and/or tethering and to decreased mitochondrial trafficking. Although the exact role of mitochondrial aggregation is unclear, it is believed that it may serve to capture these organelles at specific locations that require high energy consumption and/or high Ca^2+^ buffering conditions [8], [51]. Mitochondrial aggregating phenotypes have been observed after dysfunction of Miro and Trak2 proteins, which regulate mitochondrial trafficking. This observation suggests that the alteration of proteins regulating mitochondrial transport and trafficking may be one of the mechanisms that results in aggregation [8], [52]. Our data indicate that the mitochondrial aggregation phenotypes induced by Alex3 are dramatically regulated by the Wnt signaling cascade. Recent data have suggested several molecular links between the Wnt pathway and mitochondrial function. For instance, Wnts have been proposed to promote mitochondrial biogenesis [53], [54], increase ROS production [54], and mediate mitochondrial-induced apoptosis [55], [56]. Moreover, several downstream Wnt effectors (that interact with the Blc2/Blc-xL complex) bind to mitochondria [56], and mitochondrial-associated β-catenin has been proposed to be related to the response to leukotriene D4, to an increase in NADPH dehydrogenase activity and ATP/ADP ratio, and to the regulation of mitochondrial gene expression ROS levels [22]. Finally, mitochondrial APC has been associated with tumor survival by regulating Bcl2 [23]. Taken together with the above findings, our data underscores that the Wnt signaling cascade regulates various and convergent functions of mitochondrial biology, including the regulation of mitochondrial aggregation, dynamics and trafficking.

## Supporting Information

Figure S1 **Alex3 fully co-localizes with MitoTracker and MitoDsRed.** (**A**) The mitochondrial red marker MitoTracker (red) and the mitochondrial green marker MitoGFP (green), which shares the same mitochondrial targeting sequence of MitoDsRed (from the subunit VIII of human cytochrome c oxidase), completely co-localized in HEK293T cells. Alex3-GFP (**B,C**) and Alex3(1–106)-GFP (**D,E**) fully co-localized with both mitochondrial markers, MitoTracker (**B,D**) and MitoDsRed (**C,E**), in HEK293T cells. Scale Bar: 10 µm.
(TIF)

Figure S2 **Mitochondrial localization of Alex3 after co-expression of Wnts and Fz2.** (**A–F**) Mitochondrial localization of Alex3 (green) in HEK293AD cells after co-expression with the members of the Wnt/Frizzled signaling pathway used in Figure 3 (Wnt1, Fz2, Wnt5a and Wnt11). Alex3 protein overlaps with the mitochondrial network labeled with Mitotracker (red). (**G–L**) High magnifications of boxed areas shown in (**A–F**). Nuclei were labeled with bisbenzimide (blue). Scale bar: 10 µm.
(TIF)

Figure S3 **Alex3 does not colocalize with Frizzled2-HA.** Co-expression of Alex3 (green) and Frizzled2-HA (red) in HEK293T cells does not show colocalization. Nuclei are stained in blue (bisbenzimide). Scale Bar: 10 µm.
(TIF)

Figure S4 **Recombinant Wnt5a leads to Alex3 degradation and mitochondrial disaggregation in cells.** (**A,B**) High-magnification micrographs illustrating that Wnt5a treatment leads to mitochondrial disaggregation. (**C**) Histogram showing percentage of mitochondrial phenotypes in control and Wnt5a-treated, Alex3-transfected cells. Scale Bar: 20 µm.
(TIF)

Figure S5 **Wnt3a treatment does not reverse Alex3 mitochondrial aggregation.** Representative Alex3-transfected cells (green) treated with recombinant Wnt3a show mitochondrial aggregate phenotypes. β-catenin in red and nuclei in blue (bisbenzimide). Scale Bar: 10 µm.
(TIF)

Figure S6 **Wnt5a and Calphostin C produce Alex3 protein degradation.** Overexpressing Alex3 HEK293T cells treated with Wnt5a or Calphostin C and in presence of cycloheximide show a reduction in protein levels compared with cells treated only with cycloheximide.
(TIF)

Figure S7 **Alex3-GFP deletion constructs lacking PKC phosphorylation sites do not respond to Wnt1 and Calphostin C. (A)** Co-transfection of Wnt1 with Alex3-GFP (1–106) and (1–45) deletion constructs (right panels), which lack PKC phosphorylation sites, does not lead to Alex3 protein degradation, in comparison with co-transfection with full-length Alex3-GFP protein (left panel). (**B**) Incubation with the PKC inhibitor Calphostin C leads to degradation of full-length Alex3 (left panel) but not of Alex3-GFP (1–106) or (1–45) deletion constructs (right panel). The quantification of Alex3-GFP protein levels is shown at the bottom.
(TIF)

Video S1 Videorecording (7.5 minutes) illustrating mitochondrial dynamics in control cells.
(AVI)

Video S2 Videorecording (7.5 minutes) illustrating mitochondrial dynamics in cells transfected with Alex3-GFP.
(AVI)

Video S3 Videorecording (7.5 minutes) illustrating mitochondrial dynamics in Alex3-GFP/Wnt1.
(AVI)

Video S4 Videorecording (7.5 minutes) illustrating mitochondrial dynamics in Alex3-GFP/Wnt1 cells treated with TPA.
(AVI)
