# Supplementary figures and images for: The Non-Canonical Wnt/PKC Pathway Regulates Mitochondrial Dynamics through Degradation of the Arm-Like Domain-Containing Protein Alex3
Source: PLoS One. 2013 Jul 2;8(7):e67773. doi: 10.1371/journal.pone.0067773 (PMC3699457; doi:10.1371/journal.pone.0067773)

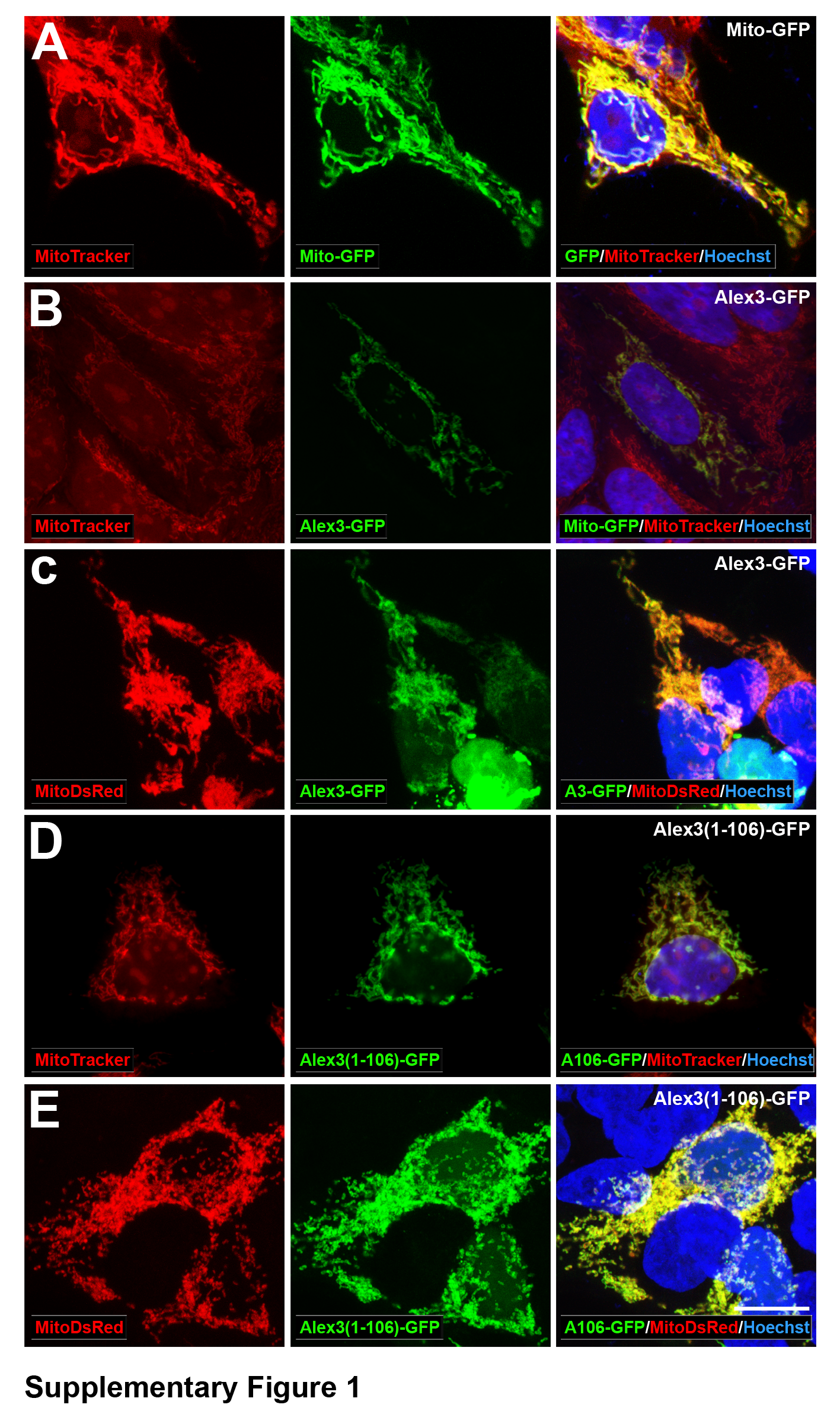

Supplement: Figure S1 — Alex3 fully co-localizes with MitoTracker and MitoDsRed. (A) The mitochondrial red marker MitoTracker (red) and the mitochondrial green marker MitoGFP (green), which shares the same mitochondrial targeting sequence of MitoDsRed (from the subunit VIII of human cytochrome c oxidase), completely co-localized in HEK293T cells. Alex3-GFP (B,C) and Alex3(1–106)-GFP (D,E) fully co-localized with both mitochondrial markers, MitoTracker (B,D) and MitoDsRed (C,E), in HEK293T cells. Scale Bar: 10 µm. (TIF) [file pone.0067773.s001.tif]

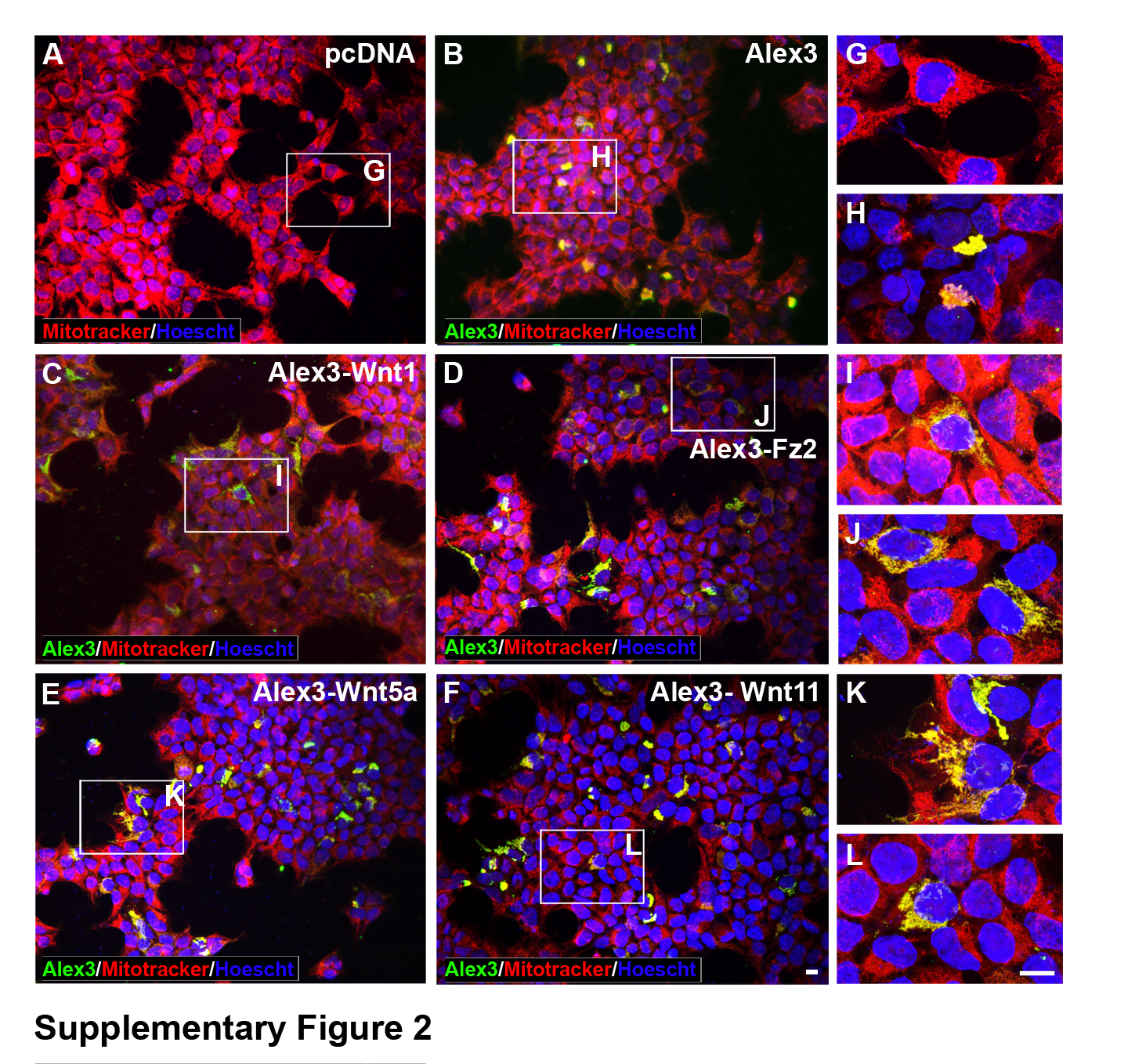

Supplement: Figure S2 — Mitochondrial localization of Alex3 after co-expression of Wnts and Fz2. (A–F) Mitochondrial localization of Alex3 (green) in HEK293AD cells after co-expression with the members of the Wnt/Frizzled signaling pathway used in Figure 3 (Wnt1, Fz2, Wnt5a and Wnt11). Alex3 protein overlaps with the mitochondrial network labeled with Mitotracker (red). (G–L) High magnifications of boxed areas shown in (A–F). Nuclei were labeled with bisbenzimide (blue). Scale bar: 10 µm. (TIF) [file pone.0067773.s002.tif]

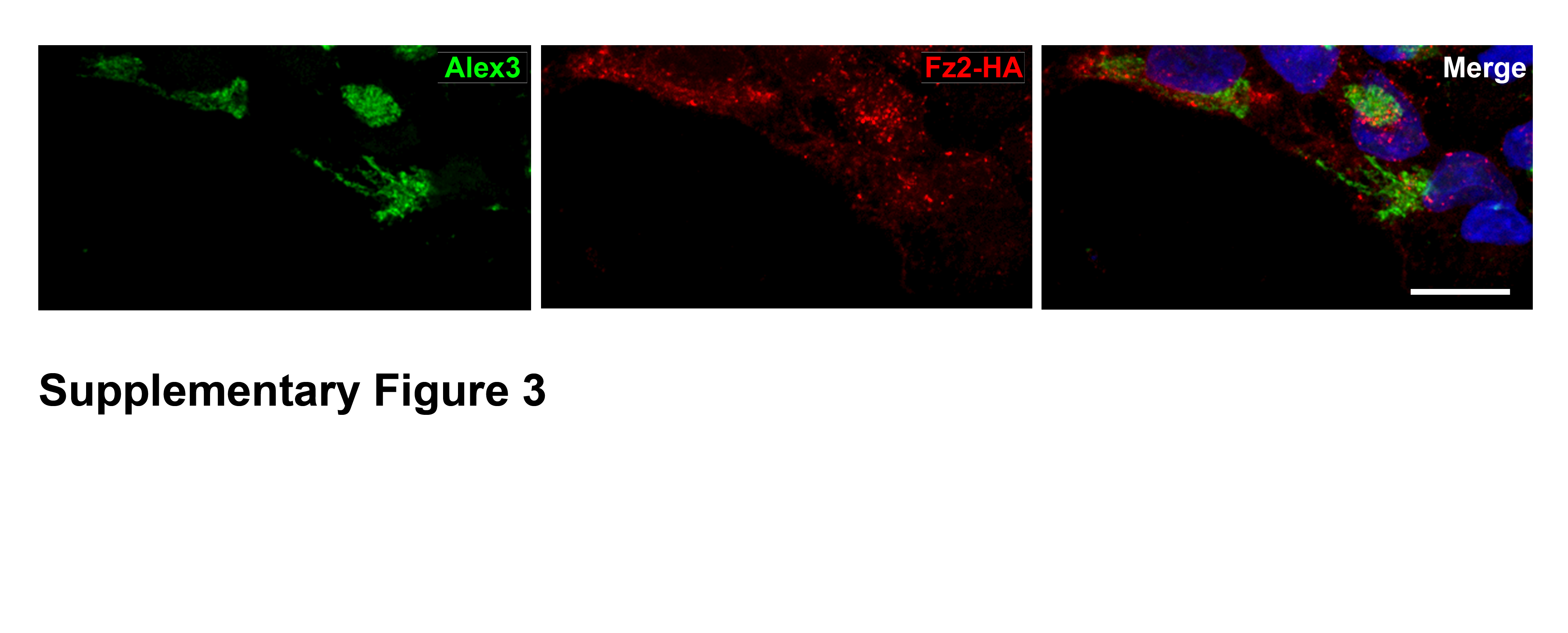

Supplement: Figure S3 — Alex3 does not colocalize with Frizzled2-HA. Co-expression of Alex3 (green) and Frizzled2-HA (red) in HEK293T cells does not show colocalization. Nuclei are stained in blue (bisbenzimide). Scale Bar: 10 µm. (TIF) [file pone.0067773.s003.tif]

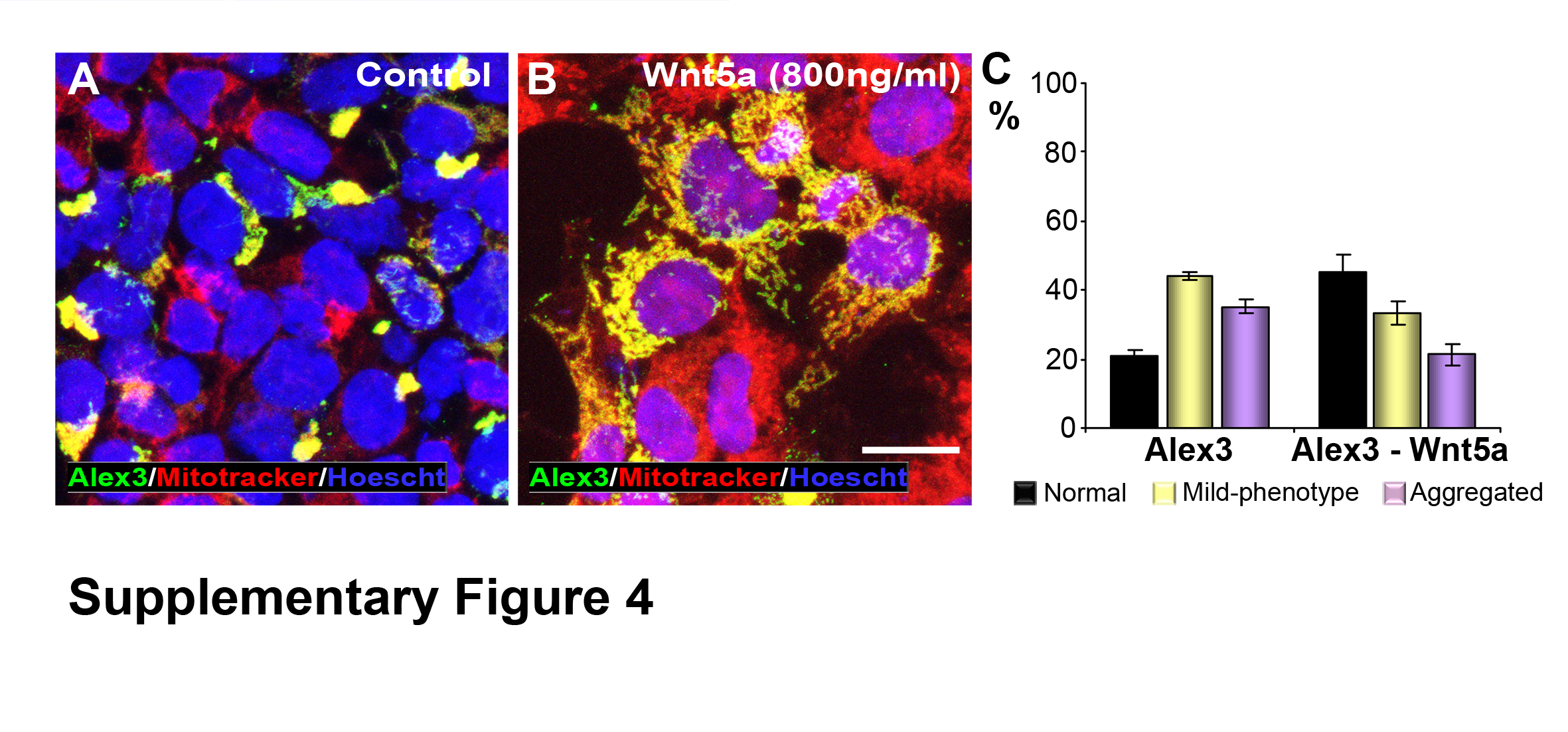

Supplement: Figure S4 — Recombinant Wnt5a leads to Alex3 degradation and mitochondrial disaggregation in cells. (A,B) High-magnification micrographs illustrating that Wnt5a treatment leads to mitochondrial disaggregation. (C) Histogram showing percentage of mitochondrial phenotypes in control and Wnt5a-treated, Alex3-transfected cells. Scale Bar: 20 µm. (TIF) [file pone.0067773.s004.tif]

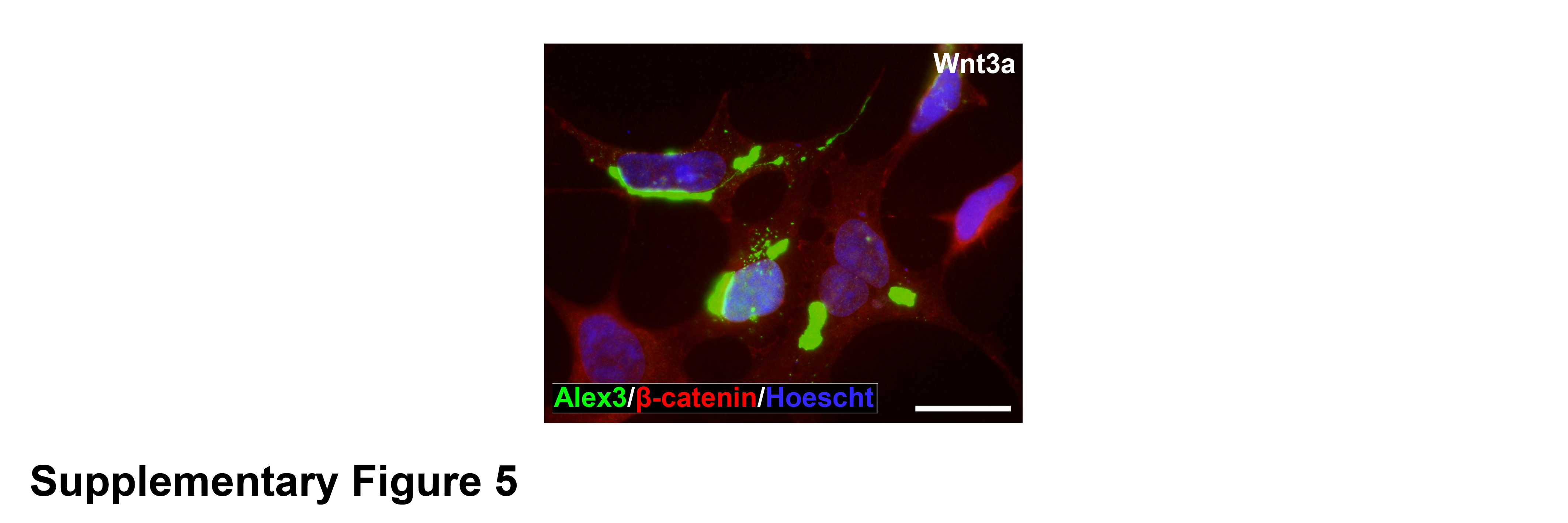

Supplement: Figure S5 — Wnt3a treatment does not reverse Alex3 mitochondrial aggregation. Representative Alex3-transfected cells (green) treated with recombinant Wnt3a show mitochondrial aggregate phenotypes. β-catenin in red and nuclei in blue (bisbenzimide). Scale Bar: 10 µm. (TIF) [file pone.0067773.s005.tif]

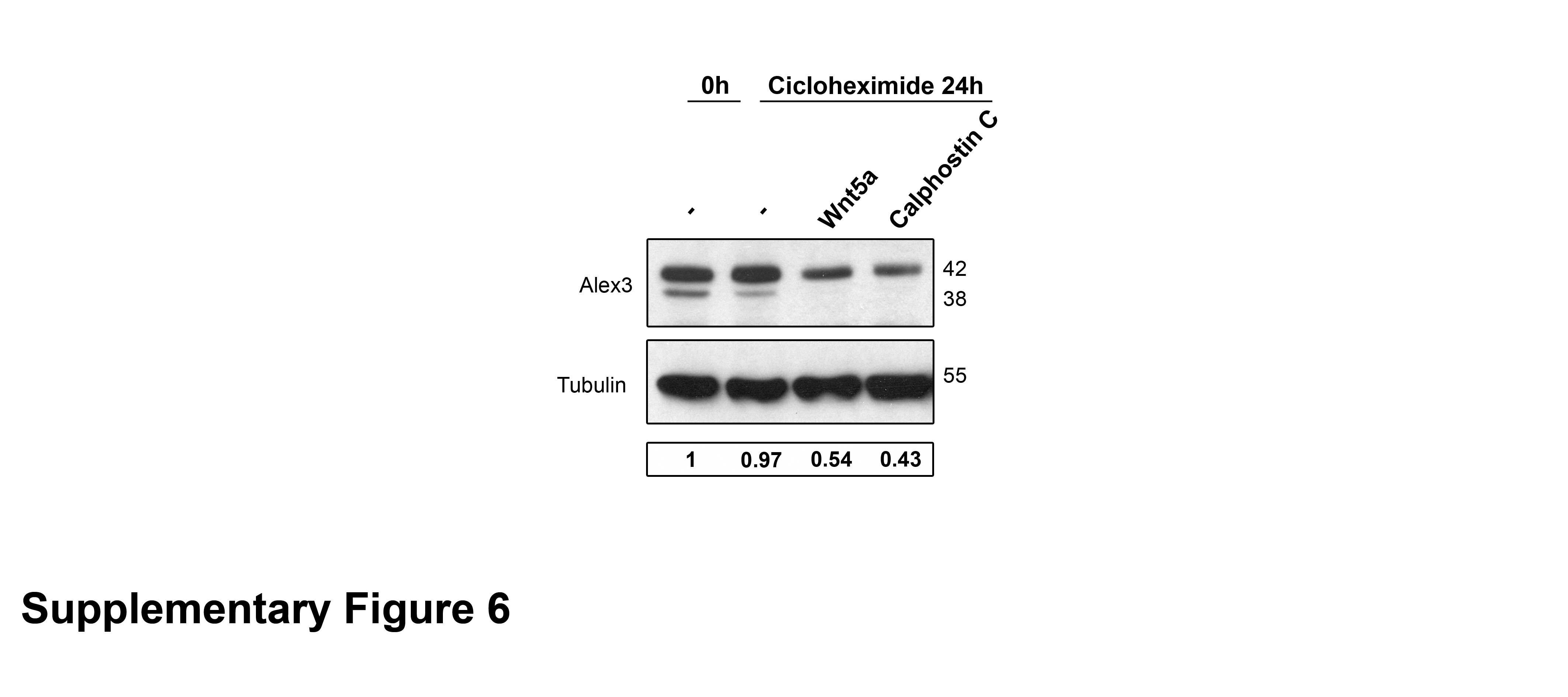

Supplement: Figure S6 — Wnt5a and Calphostin C produce Alex3 protein degradation. Overexpressing Alex3 HEK293T cells treated with Wnt5a or Calphostin C and in presence of cycloheximide show a reduction in protein levels compared with cells treated only with cycloheximide. (TIF) [file pone.0067773.s006.tif]

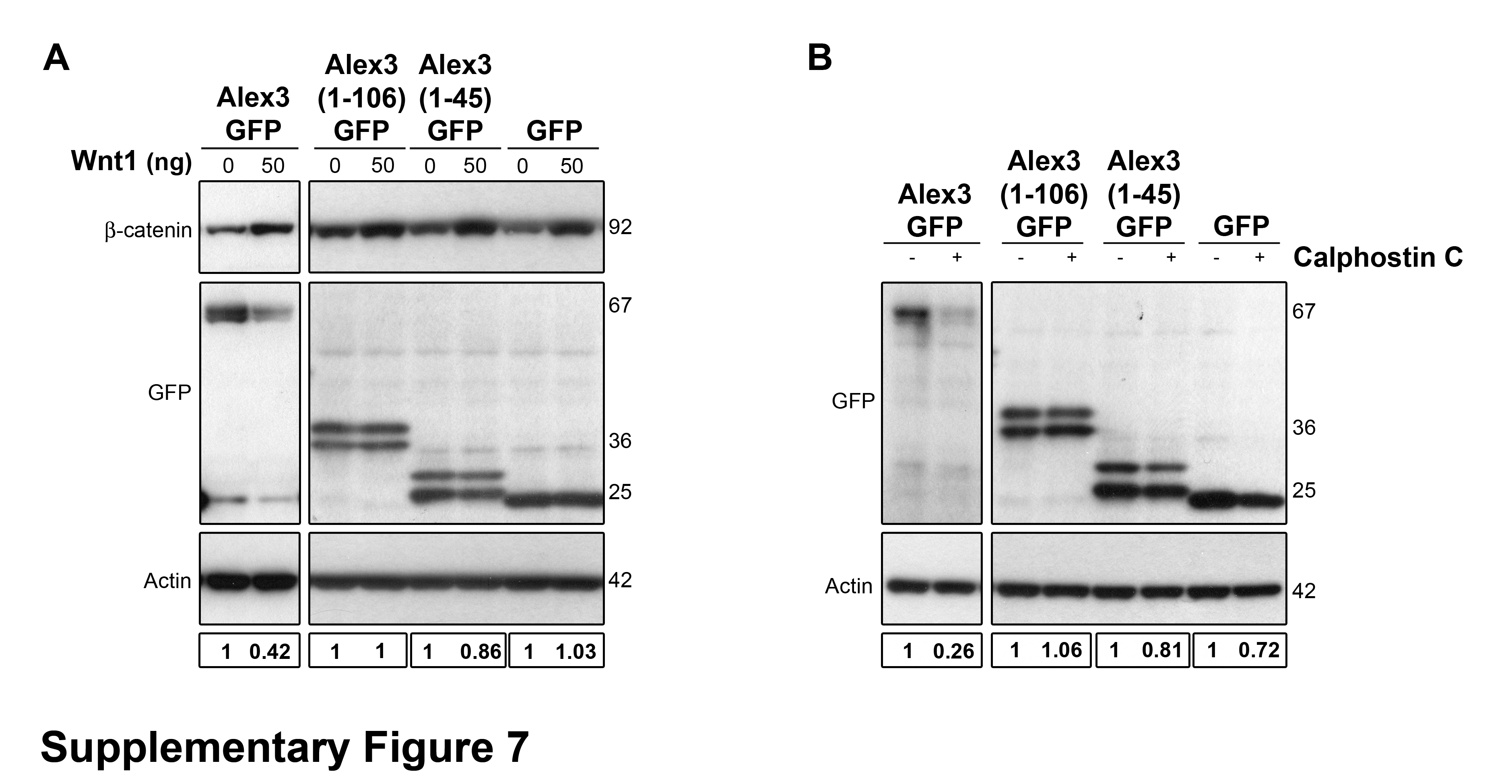

Supplement: Figure S7 — Alex3-GFP deletion constructs lacking PKC phosphorylation sites do not respond to Wnt1 and Calphostin C. (A) Co-transfection of Wnt1 with Alex3-GFP (1–106) and (1–45) deletion constructs (right panels), which lack PKC phosphorylation sites, does not lead to Alex3 protein degradation, in comparison with co-transfection with full-length Alex3-GFP protein (left panel). (B) Incubation with the PKC inhibitor Calphostin C leads to degradation of full-length Alex3 (left panel) but not of Alex3-GFP (1–106) or (1–45) deletion constructs (right panel). The quantification of Alex3-GFP protein levels is shown at the bottom. (TIF) [file pone.0067773.s007.tif]
